# Comparative Evaluation of Autologous Platelet Aggregates Versus Blood Clot On The Outcome Of Regenerative Endodontic Therapy: A Systematic Review and Meta-Analysis

**DOI:** 10.4317/jced.62508

**Published:** 2025-04-01

**Authors:** Saumya Verma, Alpa Gupta, Mrinalini Mrinalini, Dax Abraham, Unnati Soma

**Affiliations:** 1 MDS PG IIIrd year. Department of Conservative Dentistry and Endodontics, Manav Rachna Dental College and Hospital, Surajkund Badkhal Road, Sector- 43, Faridabad, Haryana, 121004; 2 Professor, MDS. Department of Conservative Dentistry and Endodontics, Manav Rachna Dental College and Hospital, Surajkund Badkhal Road, Sector- 43, Faridabad, Haryana, 121004; 3 Senior Lecturer, MDS. Department of Conservative Dentistry and Endodontics, Manav Rachna Dental College and Hospital, Surajkund Badkhal Road, Sector- 43, Faridabad, Haryana, 121004; 4 Professor, PhD. Department of Conservative Dentistry and Endodontics, Manav Rachna Dental College and Hospital, Surajkund Badkhal Road, Sector- 43, Faridabad, Haryana, 121004; 5 MDS PG IIIrd Year. Department of Conservative Dentistry and Endodontics, Manav Rachna Dental College and Hospital, Surajkund Badkhal Road, Sector- 43, Faridabad, Haryana, 121004

## Abstract

**Background:**

Regenerative endodontics represents a transformative approach to dental care, revitalizing necrotic teeth. This systematic review and meta-analysis evaluated the role of autologous platelet aggregates compared to the traditional blood clot method in regenerative endodontics.

**Material and Methods:**

A systematic search was conducted in PubMed, Scopus, EBSCO, Open Grey, and Google Scholar between 1st–12th August 2024. Case series, RCTs, retrospective studies, and case reports were included. Meta-analysis on RCTs and case series utilized RevMan 5.4 software, with *p*=0.05 as the significance level. The JBI risk of bias tool and GRADE system assessed study quality.

**Results:**

Nineteen studies met inclusion criteria, of which 13 were evaluated for risk of bias—11 showed low risk, and 2 were moderate. Rates of complete apical closure using PRF, BC, PRP, and CGF scaffolds ranged from 61.76% to 100%. Statistical analysis revealed no significant differences between autologous platelet aggregates and BC for outcomes such as complete apical closure (BC vs. PRP: *p*=0.28; BC vs. PRF: *p*=0.36), positive vitality (BC vs. PRP: *p*=0.70; BC vs. PRF: *p*=0.36), healing response (BC vs. PRF: *p*=0.23), and overall success score (BC vs. PRP: *p*=0.62).

**Conclusions:**

BC remains an effective primary scaffold for non-vital teeth with open apexes. PRP and PRF are viable alternatives when intracanal blood induction is challenging. Overall, platelet aggregates and BC showed comparable clinical and radiographic outcomes.

** Key words:**Apexogenesis, Autologous Platelet Aggregates, Blood Clot, Immature Tooth, Regenerative Endodontics.

## Introduction

Regenerative endodontics is a branch of dentistry that focuses on restoring damaged or diseased necrotic dental pulp tissues with an open apex (1). Traditionally treatment for an open apex tooth involved removing the necrotic pulp and filling the root canal with an inert material, typically gutta-percha. Procedures like apexification with calcium hydroxide or mineral trioxide aggregate (MTA) were utilized to induce apical closure, but these methods often carried a questionable prognosis since these procedures have their limitations (2). Calcium hydroxide has limited antibacterial activity, marginal leakage, greater solubility, and less cohesive strength. It leads to a reduction in root strength and increases the possibility of fracture (3,4). Mineral trioxide aggregate (MTA) is characterized by its granular consistency, which can pose challenges during insertion and packing, requiring operator expertise. Also, MTA has a long setting time and low strength, and it can be relatively expensive. While advancements have improved the handling of MTA, neither gutta-percha nor MTA can restore vitality to necrotic teeth or promote root maturation (5). Consequently, efforts to restore the vitality of necrotic teeth began, leading to the development of regenerative endodontics as a promising solution.

In regenerative endodontics, success is measured by three key outcomes: symptom elimination with bony healing (primary), 

increased root wall thickness or length (secondary), and a positive vitality test (tertiary) (6). Blood clot serves as a natural scaffold that promotes tissue regeneration within the root canal space. It contains various growth factors and cells necessary for healing, including platelets, leukocytes, and mesenchymal stem cells (7). Additionally, regenerative endodontic procedures may utilize various materials like platelet-rich plasma (PRP), platelet-rich fibrin (PRF), as well as scaffold materials such as collagen or bioceramics aiding for a conducive environment enriched with growth factors that facilitate healing and tissue formation (8). With the release of growth factors, Autologous platelet concentrates (APCs) are blood-derived substances obtained from a patient’s blood, enhancing the healing process. Activated platelets embedded in a fibrin matrix scaffold are present in these concentrates. Growth factors and cytokines secreted by APCs are critical for tissue regeneration (9). Given their ability to facilitate healing, APCs have found successful applications in both the medical and dental fraternity over the past few years. Platelet-rich plasma (PRP), platelet-rich fibrin (PRF), and concentrated growth factor (CGF) are the sources of platelets widely used in regenerative endodontics. Platelet concentrates have demonstrated several benefits over blood clots, such as the capacity to sustain growth factor levels, promote tissue regeneration, and stabilize blood clots (10). This systematic review highlights the role of autologous platelet aggregates in promoting tissue regeneration compared to blood clots within endodontic procedures. The review will evaluate various outcome parameters such as healing response, apical closure, positive vitality, and overall success score, comparing these outcomes against a control group treated with blood clots.

## Material and Methods

The review adhered to the guidelines established by Preferred Reporting Items for Systematic Reviews and Meta-Analyses (PRISMA) extension for systematic reviews. The protocol for this study was pre-registered with the International Prospective Register of Systematic Reviews (PROSPERO) before commencement (PROSPERO ID- CRD42023484150).

Information Sources.

An extensive search was carried out across clinical registry, PubMed/Medline, EBSCO, and Scopus databases from 1st August 2024 to 12th August 2024. The search was restricted to articles published between October 2012 to August 2024. A grey literature search was conducted through Google Scholar and Open Gray as well as cross-references reviewed to identify additional relevant papers for inclusion in the review.

Literature Search.

The literature search was guided by the question: How do clinical and radiographic results of regenerative endodontic treatments using autologous platelet aggregates compare to those using blood clots as scaffolds? The PICO criteria for eligibility were as follows: ( Table 1). The search strategy applied was: (((((((((((regenerative endodontics) OR (revitalization)) AND (revascularisation)) OR (platelet concentrates)) OR (autologous platelet concentrates)) OR (autologous aggregates)) OR (scaffolds)) OR (PRP)) OR (PRF)) AND (BC)) OR (Blood clot)) AND (Permanent Tooth).

The reference lists of qualifying studies were reviewed to find additional pertinent research, and active studies were explored through clinical trial registries. Two authors, SV and AG, independently carried out the literature search following the predefined strategy. They also independently evaluated the studies for inclusion and conducted data extraction. In cases of any conflict, a third expert, DA, was sought to resolve discrepancies.

Study Selection and Eligibility Criteria

Inclusion Criteria:

Studies comparing blood clots with PRP or PRF or CGF, studies done on permanent teeth with immature or open apices, Randomized control trials/ Non- Randomised Control trials/ case studies/ case report/ retrospective studies, and studies with a 12-month follow-up for assessing outcomes after RET.

Exclusion Criteria:

Studies without a Blood clot group as a control group, Animal studies/ *in vitro* studies, Studies conducted on permanent teeth with Mature apices or on primary teeth, Articles for which the complete text was unavailable or the material and method section was poorly described; though the authors were contacted prior to exclusion and articles in a language other than English

Data Collection Process:

A tailored spreadsheet was crafted to streamline data collection from the selected studies. Two calibrated reviewers (SV and AG) extracted information, which included author names, journal sources, study design, number of teeth or patients, group classifications, types of scaffolds used, induced bleeding, external scaffolds applied, coronal sealing materials near the scaffold, follow-up protocols, clinical success, root length and width changes, closure of apex, radiographic software used for assessment, pulp sensibility, and any other relevant outcomes.

This review’s objective is to compare the clinical success rates of autologous scaffolds used in regenerative endodontic treatment (RET) to blood clots as scaffolds. It also evaluates healing response, response to vitality, and achievement of apical closure following RET. The Cohen’s kappa values among the examiners varied from 0.73 to 0.83 for the various collected variables.

Risk Of Bias:

The risk of bias was evaluated utilizing Joanna Briggs Institute critical appraisal tool for systematic reviews. The included studies consisted of randomized controlled trials and case series. For randomized controlled trials, 13 domains were assessed, including randomization, allocation concealment, baseline similarity of groups, participant blinding, operator blinding, treatment received, outcome assessor blinding, similarity of outcome assessment, outcome reliability, follow-up, participant analysis in randomized groups, statistical analysis, and trial design. The risk of bias was assessed using the Joanna Briggs Institute critical appraisal tool for the case series where 10 domains were evaluated. The evaluation process was conducted independently by two reviewers, SV and AP. In case of a conflict, a third reviewer, DA, was sought, and their decision was deemed final.

Data Synthesis and Analysis:

The success outcomes were compiled from the individual study results and shown as a range, accompanied by the overall sample size employed to assess each outcome. Statistical heterogeneity among studies was assessed using Risk of Bias (ROB) analysis, the chi-square (χ²) test, and I² statistics. A pairwise meta-analysis was conducted for studies that included at least two trials directly comparing different scaffolds for clinical success, employing a random-effects model. Quantitative analysis was performed using Review Manager Software 5.4, with forest plots created to evaluate overlapping results across studies. Risk ratios were calculated with a fixed-effect model to compare clinical success between PRP vs. BC and PRF vs. BC. Cohen’s kappa statistics were utilized to evaluate inter-reviewer agreement, with statistical significance established at *p*<0.05.

Certainty of Evidence:

Using the GRADE (Grading of Recommendations, Assessment, Development, and Evaluations tool) [Software], an assessment of the evidence of the meta-analysis results was evaluated (Available from https://www.gradepro.org/). GRADE was applied only to the RCTs included in the meta-analysis. This grading method evaluates five dimensions that have the potential to reduce the degree of certainty in the evidence: indirectness, imprecision, consistency, risk of bias, and additional considerations such as publication bias, effect size, plausible confounding factors, and dose-response gradient.

**Figure 1:**
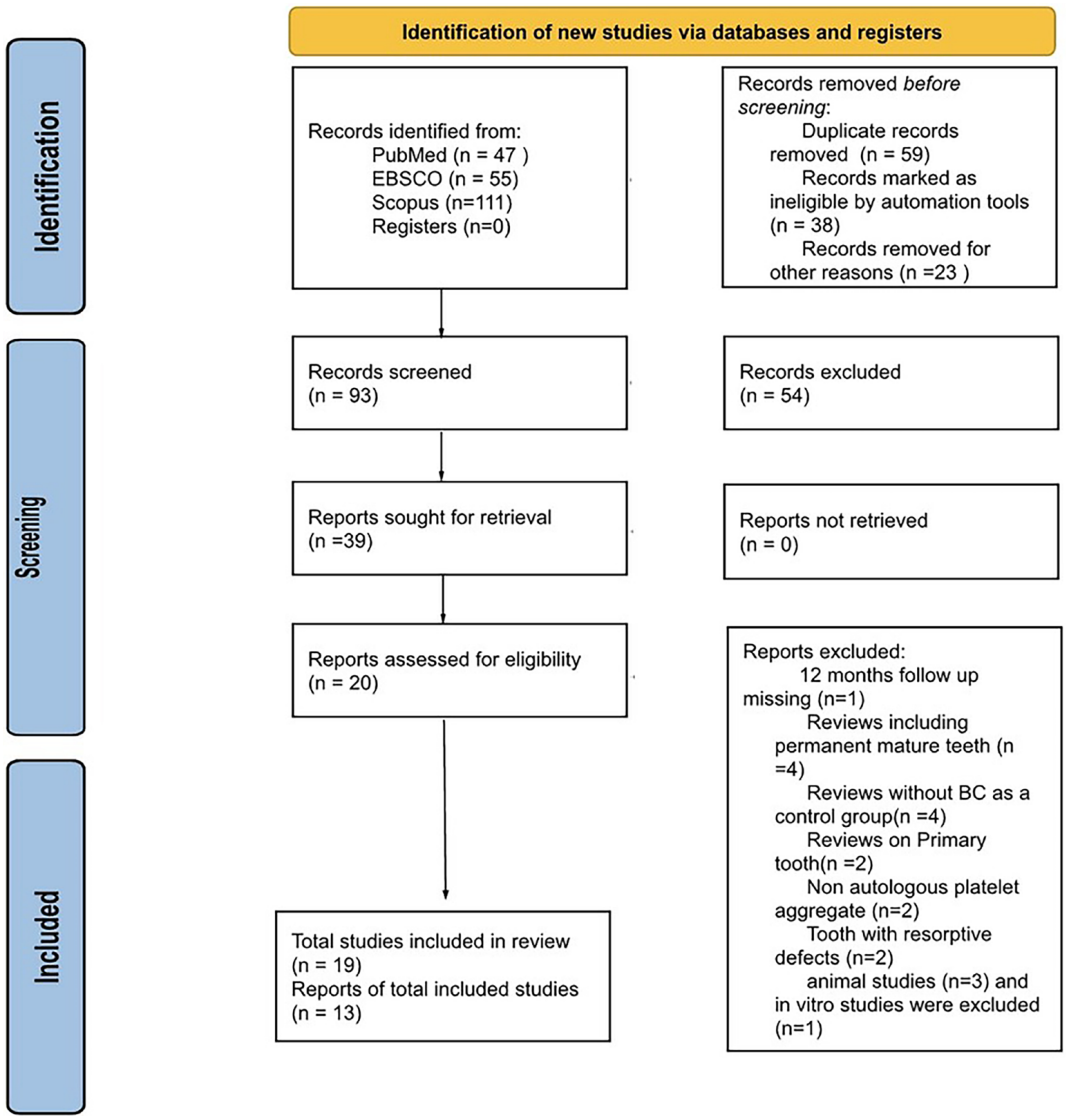
A flowchart of the screening of studies according to the Preferred Reporting Items for Systematic Reviews and Meta-analyses Recommendation.

RESULTS- Study Selection:

A comprehensive search of the databases and registries yielded a total of 213 articles. Thirty-nine full-text articles were shortlisted after screening and removal of duplicates. The shortlisted articles were subjected to additional eligibility evaluation, leading to the inclusion of 19 articles for reliability assessment, as well as qualitative and quantitative analysis. The details of the search process are presented in a Preferred Reporting Items for Systematic Reviews and Meta-analysis (PRISMA) flowchart (Fig. 1).

Study Characteristics:

Among the 19 studies included in the systematic review, 11 were randomized controlled trials (RCTs) (11-21), 5 were case series (22-26), 2 were retrospective studies (27,28), and 1 was a case report (29).

Table 2 provides a comprehensive overview of the demographic information and characteristics of the studies included, encompassing author names, publication years, journal sources, study types, patient numbers, groups, scaffolds utilized, outcomes measured, as well as inclusion and exclusion criteria.

**Figure 2:**
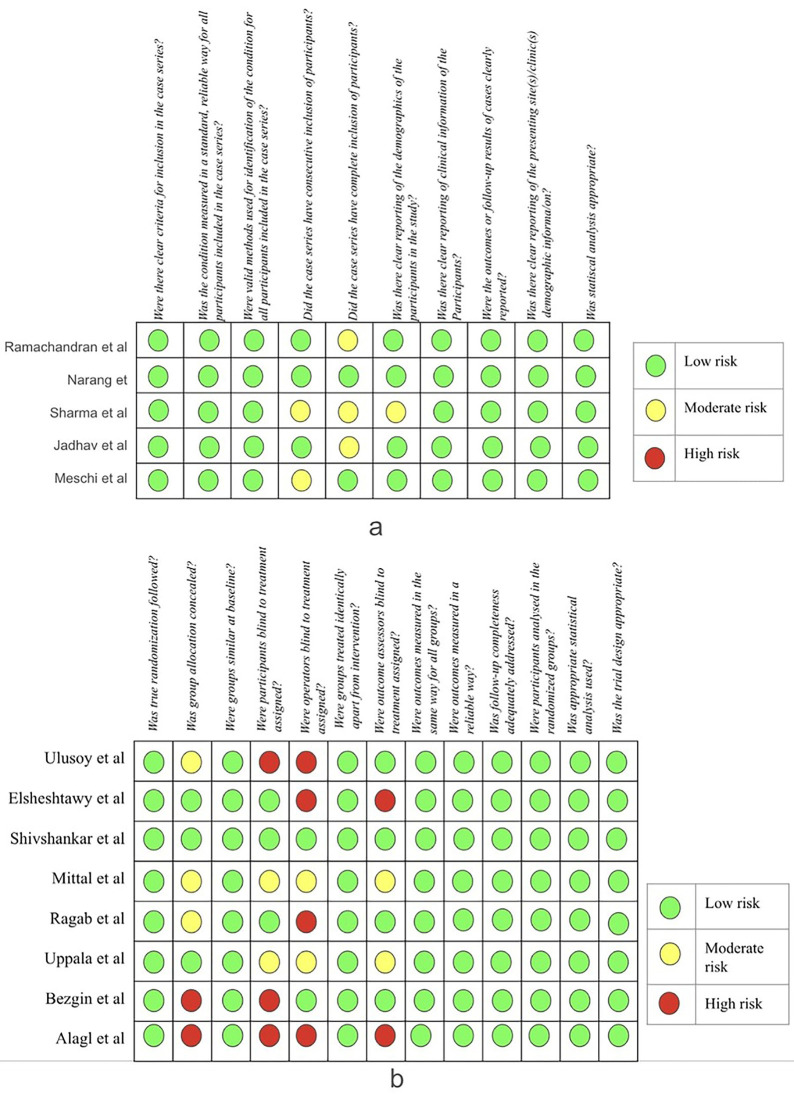
a. JBI critical appraisal for Case series. b. JBI critical appraisal for RCTs.

Quality Evaluation Of Individual Studies Based On The Study Design and Risk Of Bias:

The risk of bias was evaluated using the JBI critical appraisal tool for systematic review. The study design comprised randomized controlled trials and case series. Each study was evaluated per its specific study design. (Fig. 2.a,b) Except for two studies (18,14), all others exhibited a low risk of bias.

Comparative Assessment Of Outcome Through Qualitative analysis of various Scaffolds:

The findings of the chosen studies were represented as ranges alongside the respective sample sizes employed to ascertain specific success outcomes. Data concerning the primary outcome, namely complete apical closure after regenerative endodontic therapy (RET) utilizing various scaffolds, as well as other outcome measures like success score, pulp vitality, and periapical healing post-RET using diverse scaffolds, were extracted from the studies included. The rates of complete apical closure following RET utilizing PRF, BC, PRP and CGF as intracanal scaffolds varied between 64.64%–100%, 61.76%–100%, 70.64%–100% and 86%–91% respectively.

Quantitative Analysis for Individual Studies

Meta-analysis.

All meta-analyses were carried out using RevMan 5.4 software, with random risk ratios computed using the Mantel-Haenszel method. Data from the studies were analyzed and categorized into 2 groups depending on the effect size characteristics.

**Figure 3:**
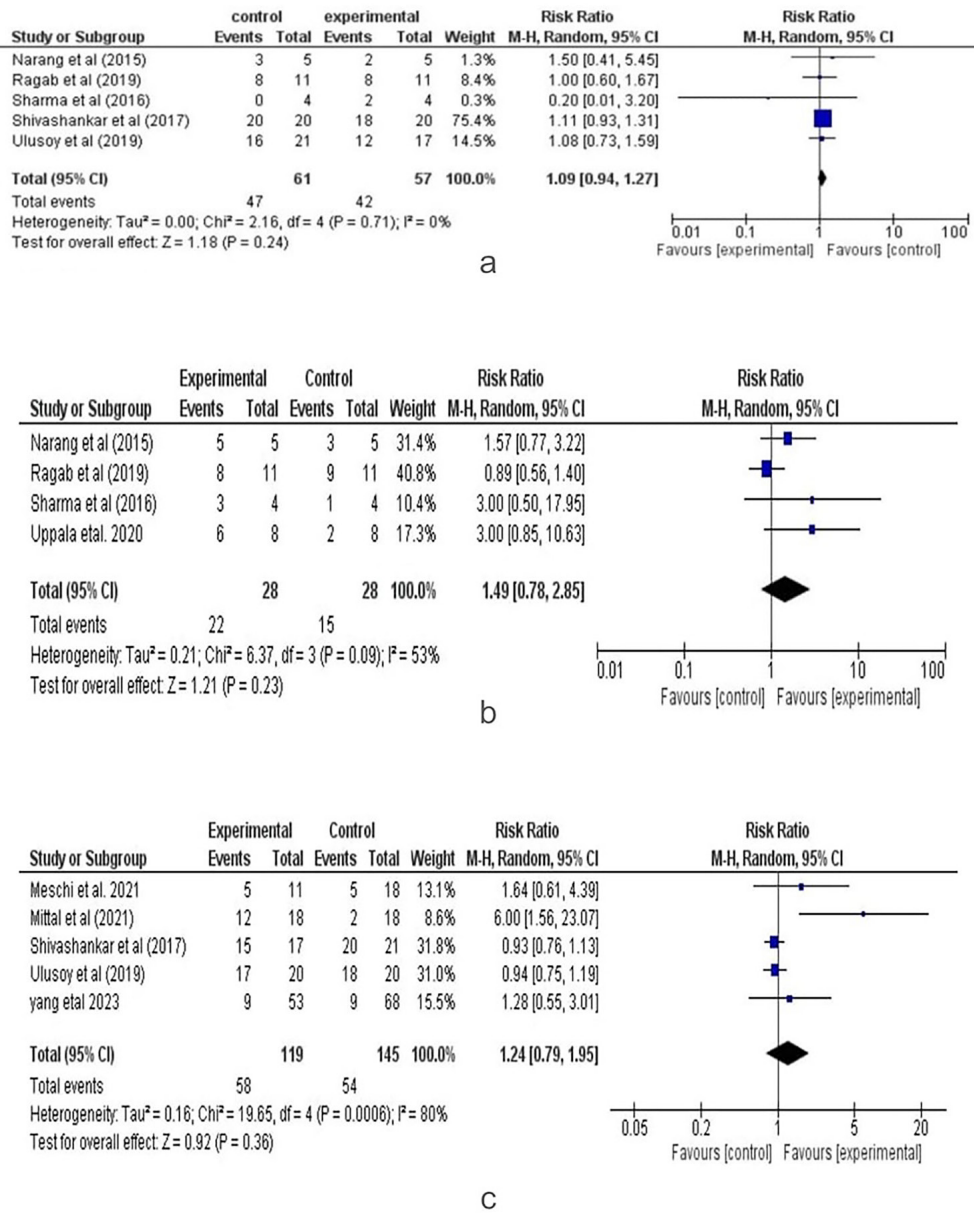
a. Complete Apical Closure (BC vs PRF) Forest Plot. b. Healing Response (BC vs PRF) Forest Plot. c. Positive Vitality (BC vs PRF) Forest Plot.

Complete Apical Closure (BC vs PRF): Six studies assessed complete apical closure, with a dichotomous (binary) effect size. The analysis focused on comparing the Blood Clot (control group) with PRF (experimental group) for complete apical closure in regenerative endodontic procedures. Figure 3.a presents the forest plot from the random-effects meta-analysis. The combined risk ratio was 1.10 (95% CI: 0.95, 1.27), indicating a result favoring the control group, though the difference was not statistically significant (*p*=0.21). Statistical heterogeneity was low (I²=0%), and the GRADE quality of evidence was rated as high.

Healing Response (BC vs PRF): Four studies assessed healing response, with a dichotomous (binary) effect size. The analysis focused on comparing the Blood clot (control group) and PRF (experiment group) for healing response in regenerative endodontic procedures. Figure 3.b presents the forest plot from the random-effects meta-analysis. The combined risk ratio was 1.49 (95%, CI: 0.78, 2.85), indicating a result favoring the control group, though the difference was statistically insignificant (*p*=0.23). Statistical heterogeneity was moderate (I2=53%), and the GRADE quality of evidence was rated as moderate.

Positive Vitality (BC vs PRF): Five studies assessed positive vitality, with a dichotomous (binary) effect size. The analysis compared the Blood clot (control group) with PRF and CGF (experiment groups) regarding positive vitality tests in regenerative endodontic procedures. Figure 3.c presents the forest plot from the random-effects meta-analysis. The combined risk ratio was 1.24 (95% CI: 0.79, 1.95), indicating a result in favor of the experimental group, though the difference was statistically insignificant (*p*=0.36). Statistical heterogeneity was high (I2=80%), and the GRADE quality of evidence was rated as high.

**Figure 4:**
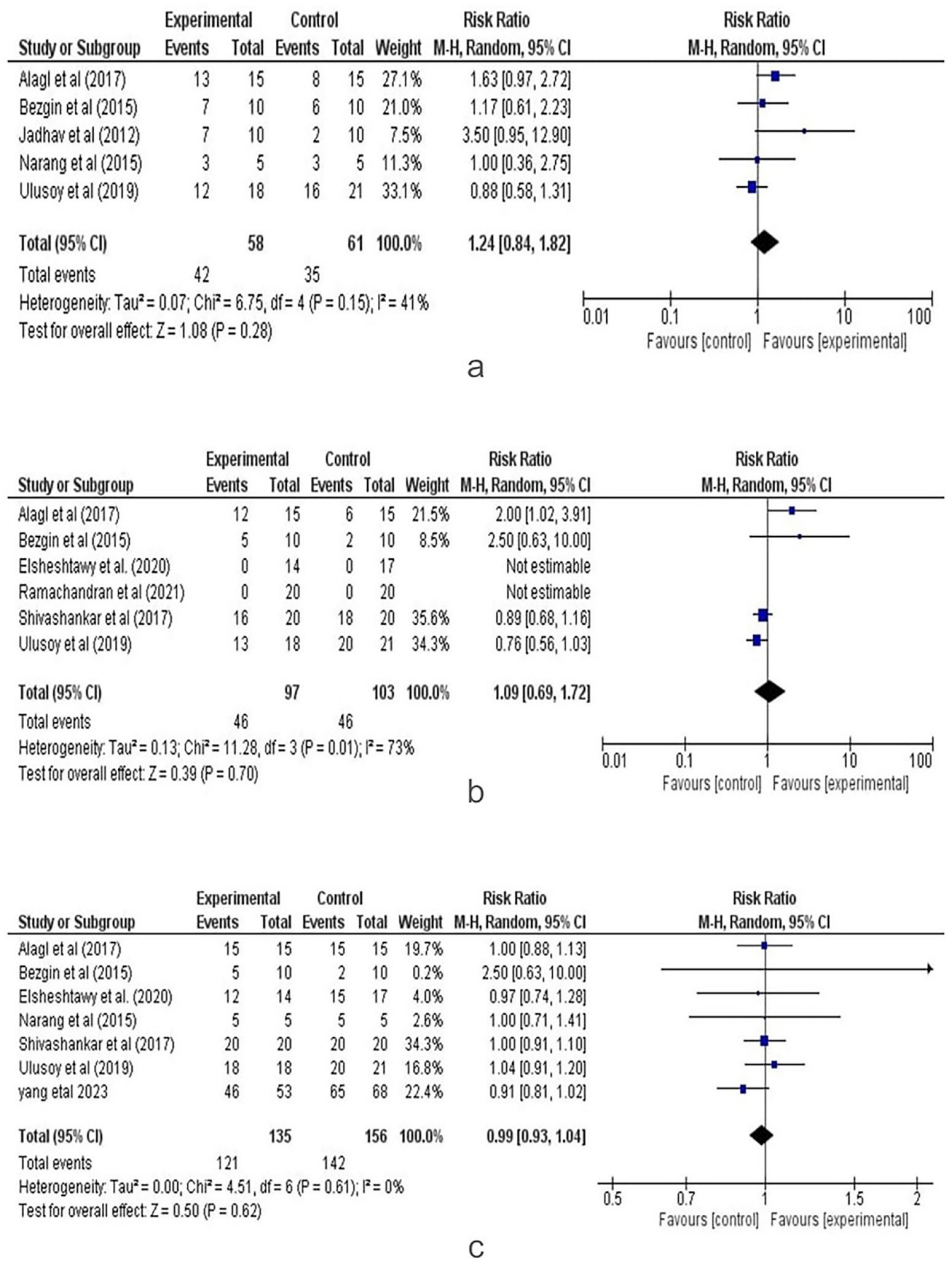
a. Complete Apical Closure (BC vs PRF) Forest Plot. b. Healing Response (BC vs PRF) Forest Plot. c. Positive Vitality (BC vs PRF) Forest Plot.

Complete Apical Closure (BC vs PRP): Five studies assessed complete apical closure with a dichotomous (binary) effect size. The analysis compared the Blood clot (control group) with PRP (experiment group) in terms of complete apical closure in regenerative endodontic procedures. Figure 4.a presents the forest plot from the random-effects meta-analysis. The combined risk ratio was 1.24 (95% CI: 0.84, 1.82), indicating a result favoring the experiment group, though the difference was statistically insignificant (*p*=0.28). Statistical heterogeneity was moderate (I2=41%), and the GRADE quality of evidence was rated as high.

Positive Vitality (BC vs PRP): Six studies assessed positive vitality with a dichotomous (binary) effect size. The analysis compared the Blood clot (control group) with PRF (experiment group) in terms of positive vitality tests in regenerative endodontic procedures. Figure 4.b presents the forest plot from the random-effects meta-analysis. The combined risk ratio was 1.09 (95% CI: 0.69, 1.72), indicating a result favoring the experiment group, although the difference was statistically insignificant (*p*=0.70). A substantial degree of statistical heterogeneity was observed among the included studies(I2=73%), and the GRADE quality of evidence was rated as moderate.

Success Score (BC vs PRP): Seven studies assessed success scores with a dichotomous (binary) effect size. The analysis compared the Blood clot (control group) with PRF and CGF (experiment groups) in terms of success scores in regenerative

endodontic procedures. Figure 4.c presents the forest plot from the random-effects meta-analysis. The combined risk ratio was 0.99 (95% CI: 0.93, 1.04), favoring the experiment group, though the difference was not statistically significant (*p*=0.62). Statistical heterogeneity was low among the included studies (I2=0%), and the GRADE quality of evidence was rated as moderate.

GRADE (Grading of Recommendations, Assessment, Development, and Evaluations tool):

The GRADE assessment indicated a moderate to high level of certainty regarding the evidence for the measured outcomes. The overall quality of evidence between the success score of BC and PRP is downgraded because one of the studies had a high risk of bias. For complete apical closure, between BC/ PRP and BC/PRF, the studies have a high certainty of evidence. The overall positive vitality between BC and PRF has a high certainty of evidence. The certainty of evidence for positive vitality between BC and PRP is moderate. The certainty of evidence for healing response in BC and PRF groups is downgraded to moderate due to serious inconsistency and imprecision (See explanation in  Table 3 for explanations).

## Discussion

Regenerative endodontic treatment, performed in immature permanent teeth, prioritizes the revascularisation of the dental pulp, yielding outcomes like complete apical closure and pulp vitality restoration (30). The rationale for regenerative endodontic treatment is the remarkable resilience of apical papilla tissue, which can withstand adverse conditions and regenerate under favorable conditions after the reduction of infection load (31). An ideal scaffold for regenerative procedures should be biocompatible, and biodegradable, facilitate cell binding and localization, and supply growth factors. Autologous platelet aggregates, which include BC, PRF, PRP, and CGF, are the most frequently used scaffolds for regenerative endodontic treatment due to their idealist properties (32). Various synthetic scaffolds are also employed for this procedure, including poly-lactic-co-glycolic acid membrane, chitosan, collagen membrane, and platelet pellet, all of which have shown a 100% success rate (33). Blood clot formation is a well-understood physiological process that normally takes place in the event of tissue damage to achieve hemostasis and initiate the healing process for wounds (34). The use of BC as scaffolds in RET has been reported to be quite successful and feasible (35). Through a systematic evaluation of the relevant literature, the review focuses on the effects of various autologous platelet aggregates used as scaffolds in comparison to the blood clot, serving as the control group, on RET outcomes. An electronic data search was done from 2012 to 2024 to determine how various intracanal scaffolds affect treatment outcomes for regenerative endodontic therapy (RET), including full apical closure, clinical success, radiographic success, and tooth sensibility. All the included studies were diagnosed with pulpal necrosis with immature root and periapical pathology. Predominantly, the studies in this systematic review comply with the clinical guidelines for regenerative endodontic procedures outlined by the American Association of Endodontists. A systematic review that included data from 2008 to 2021—including research from 2008 to 2021—was published in February 2023; however, not all of the studies included blood clots (BC) as a control group. Moreover, the study characteristics did not include case reports and case series that compared BC with other autologous platelet aggregates (33). The results for apical root closure were statistically non-significant across different autologous scaffolds compared to BC. There is published literature to support this outcome that the stem cell population in BC is greater than found in PRF and PRP, which is derived from peripheral blood. However, PRF and PRP (concentration five times higher than normal platelet count) contain concentrated platelets, which continuously release various growth factors, aiding in tissue regeneration (25). Even with low growth factors, the results with BC were comparable with those of other autologous platelet aggregates because of an increased number of stem cells present in the periapical area (36). The healing response with BC was comparable to that with other platelet aggregates and showed nonsignificant results. However, healing was reported to be faster with PRF. PRF, with a 210-fold higher concentration of platelets, makes an even more potent scaffold, ideal for RET (36). In addition, PRF aids in reducing infection and inflammation because it contains leukocytes, cytokines, and some lymphocytes (37). When BC and other autologous platelet aggregates were evaluated, the outcome of positive pulp vitality was not statistically significant. However, compared to the BC group, PRP and PRF displayed a noticeably quicker initial response to vitality tests, which would point to a higher level of organization in the vital pulp tissue (11). The overall success score among the autologous platelet aggregate and BC in RET was statistically nonsignificant. This can be due to the data indicating that PRP or PRF fails to exhibit an improvement over BC in clinical success in cases of necrotic immature teeth indicating that BC may be equally effective for RET (38). The outcomes of this meta-analysis are non-significant, as scaffolds derived from the patient’s circulating blood, despite being anticipated to be rich in platelets and growth factors, do not demonstrate a noTable advantage over the blood clot. The current systematic review and meta-analysis have assessed the importance of various autologous platelet aggregates used as scaffolds in RET. However, determining the most effective scaffold proves challenging based on the observed outcomes in the current research. Variability in sample sizes between groups diminishes the reliability of comparisons. Heterogeneity in assessment methods for complete apical closure and healing response following RET prevents quantitative analysis. Similarly, qualitative analysis on pulp sensibility response after RET, as a successful outcome, is limited by few studies with diverse scaffold comparisons. Less commonly used scaffolds cannot be compared due to small sample sizes and limited studies.

## Conclusions

BC, PRP, and PRF are the most widely used scaffolds for regenerative endodontic treatment. Overall, success in regenerative endodontic treatment (RET) with various scaffolds is notably high, with no significant impact observed based on scaffold type. No statistically significant difference was observed for different outcome parameters such as apical root closure, positive pulp vitality, healing response, and overall clinical success when blood clot was compared to PRP and PRF. Therefore a conclusion can be drawn that BC serves as an effective primary scaffold in RET for non-vital immature teeth, reserving PRP and PRF for cases where difficulty to induce intracanal blood is encountered.

## Figures and Tables

**Table 1:** PICO.

PICO	Under study
Population	Patients with Immature Permanent Teeth
Intervention	Experimental group utilizing autologous platelet aggregates (e.g., PP, PRP, PRF, CGF, or LPRF) as scaffolds in regenerative endodontic procedures
Comparison	Control group utilizing blood clot as scaffold in regenerative endodontic procedure.
Outcome	Clinical and radiological indicators- Success Score, Apical Closure, Positive Vitality, Healing response

PICO: Population, Intervention, Comparison, Outcome, PP: Platelet pellet, PRP: Platelet rich plasma, PRF: Platelet rich fibrin, CGF: Concentrated growth factor, LPRF: Leukocyte- and Platelet-rich fibrin.

**Table 2:** Comprehensive overview of the demographic details and characteristics of the included studies.

Author	Journal	Type of Study	No.of patients	Groups	Scaffold	Outcome
Ulusoy et al., 2019 (11)	Journal of Endodontics	RCT	88 teeth in 77 participants	4	PRF, PRP, BC, PP	Clinical: Follow up every 3 months in the first year and then annually. 76 teeth (86%) showed positive response to sensitivity tests in all groups. Radiographically: follow-ups every 6 months in the first year and then annually. 73.9% (n=54) teeth showed complete apical closure. Out of these 54 teeth, 41 (75.9%) teeth had natural conical-shaped root apex and 13 (24.1%) teeth had blunt apex. 4.1% (n=3) teeth had ongoing apical closure. 21.9% (n=16) teeth showed no signs of root development. Radiographically: Rate of complete apical closure in the groups, PP (82.4%), BC (76.2%), PRF (70.6%), PRP (66.7%). On 27 months follow-up, all teeth showed similar results for RRA and RCA.
Elsheshtawy et al.,2020 (12)	International Endodontics Journal	RCT	26 participants Mean age 12.66 ±4.47	2	PRP, BC	Clinically: favorable clinical outcomes. Patients experienced reduced symptoms and improved pulp vitality, indicating successful regeneration of pulp tissue. High patient satisfaction was reported with the treatment. Radiological Outcomes: The study assessed the effects of PRP as a scaffold in regeneration/revitalization endodontics using 2-dimensional radiographs and cone beam computed tomography (CBCT). The radiological assessment revealed significant improvements in periapical healing, root lengthening, and canal wall thickening. The CBCT images displayed evidence of successful tissue regeneration and dentin formation, further supporting the efficacy of PRP in endodontic treatments.
Jadhav et al.,2012 (25)	Journal of Endodontics	Clinical Study	20 participants Age: 15-28	2	PRP, BC	Clinical outcomes: demonstrated all cases were asymptomatic with complete resolution of signs. Radiological outcomes: PRP (group 2) showed significant improvement in periapical healing, apical closure, and dentinal wall thickening compared to BC (group 1). However, root lengthening was comparable between both groups.
Meschi et al., 2021 (26)	Journal of Endodontics	Multicentre controlled clinical trial	23 teeth (9 test 14 control)	2	REP+ LPRF test group & REP- LPRF control group	Clinical Outcomes: Three teeth in the test group experienced flare-ups within the first year post-REP. Most analyzed teeth survived up to three years post-REP, with apexification used in failed cases. Qualitative assessment showed 55.6% of test group teeth had no root development or apical closure. Radiological Outcomes: PR evaluation found 91.3% and 87% achieving complete periapical bone healing (PBH) qualitatively and quantitatively, respectively, with no baseline differences between groups. However, CBCT showed only 50% of assessed teeth had complete PBH. Three years post-REP, volumetric measurements favored the control group in root hard tissue volume (p=0.03), mean thickness (p=0.003), and apical area (p=0.05). No significant differences were found in root lengthening (p=0.72) or maximum thickness (p=0.4). The correlation between PR and CBCT variables was weak to very weak.
Rizk et al.,2019 (19)	International Journal of paediatric dentistry	RCT	26	2	PRP, BC	Clinical outcomes: PRP-treated teeth showed no response to sensibility tests after 12 months, while BC-treated teeth exhibited significantly higher crown discoloration compared to PRP. Radiological outcomes: PRP-treated teeth demonstrated a statistically significant increase in radiographic root length, width, and periapical bone density, along with a decrease in apical diameter compared to BC-treated teeth.
Alagl et al., 2017 (18)	Journal of medical research	RCT	15 participants (30 teeth)	2	PRP, BC	Clinical outcomes: The study suggests that PRP demonstrates success as a scaffold for regenerative endodontic treatment. However, aside from significantly increased root length, PRP did not yield outcomes significantly different from those using blood clot as the scaffold. Radiological outcomes: PRP-treated teeth exhibited statistically significant increases in root length and width, periapical bone density, and reductions in apical diameter compared to those treated with BC. Additionally, all treated teeth did not respond to sensibility tests after 12 months, and BC showed significantly higher crown discoloration than the PRP group.
Bezgin et al.,2015 (17)	Journal of Endodontics	RCT	20 participants 22 teeth	2	PRP, BC	Clinical outcomes: PRP as a scaffold demonstrated promising clinical outcomes, including symptom resolution and positive pulp responses. It showed successful treatment for immature teeth. Radiological outcomes: Radiographically, PRP resulted in root development, periapical healing, and increased root length compared to conventional treatments.
Shivshankar et al.,2017 (13)	Journal of clinical and diagnostic research	RCT	60 participants	3	PRP, BC, PRF	Clinical outcomes: PRP, PRF, and induced bleeding demonstrated successful symptom resolution and positive pulp responses in teeth with necrotic pulp and open apex. Patients showed improvement in tooth vitality and reduced symptoms. Radiological outcomes: All three approaches (PRP, PRF, induced bleeding) resulted in comparable outcomes for root development and periapical healing. There were no significant differences observed in radiographic measures such as root lengthening or periapical bone healing among the groups.
Mittal et al.,2021 (14)	Indian Journal of Dental Research	RCT	36 teeth	4	BC, PRF, collagen, hydroxyapatite	Clinical outcomes: Pulp sensibility testing showed non-significant results across all intervals for all treatment groups (PRF, collagen, hydroxyapatite, periapical bleeding). Positive responses to the cold test were observed in varying percentages over time, with PRF demonstrating the highest responses at 12 months (66.6%). However, none of the groups showed positive responses to heat and electric pulp testing. All groups exhibited good periapical healing at the end of 12 months. Radiological outcomes: Radiographically, all treatment groups (PRF, collagen, hydroxyapatite, periapical bleeding) showed good periapical healing at the end of 12 months. However, specific differences in root development or other radiographic measures were not reported in the provided information.
Ragab et al., 2019 (15)	The Journal of Clinical Pediatric Dentistry	RCT	22 teeth	2	BC, PRF	Clinical outcomes indicated significant improvement in symptoms across both BC and PRF groups. However, there were no notable differences in positive pulp responses between the two groups. Radiological outcomes: Most cases in both groups demonstrated evidence of periapical healing, often accompanied by the formation of calcific bridges at the cervical and/or apical regions. These findings suggest comparable effectiveness between BC and PRF as scaffolds in regenerative endodontic procedures, with no significant differences observed in radiographic outcomes after 12 months.
Sharma et al., 2016 (24)	Saudi dental Journal	Clinical Study	16 cases	2	BC, PRP,PRF, BC+Collagen, BC+PLGA	Clinically, patients were completely asymptomatic throughout the study period. Radiological outcomes: All cases showed improvement in terms of periapical healing, apical closure, root lengthening, and dentinal wall thickening. PRF and collagen gave better results than blood clot and PLGA in terms of periapical healing, apical closure, and dentinal wall thickening
Rizk et al.,2020 (20)	International Journal of paediatric dentistry	RCT	24 teeth	2	BC, PRF	Clinical Outcomes: At the end of the follow-up period, all treated teeth were negative to the sensibility test. Blood clot displayed greater crown discoloration in comparison to PRF group. Radiological outcomes: Root length and width, increased periapical bone density, and a reduction in apical diameter when compared with BC
Narang et al., 2015 (23)	Contemporary clinical dentistry	Clinical Study	20 participants	4	MTA, PRF, PRP, BC	Clinical evaluation considered relief from pain, absence of swelling, drainage and resolution of sinus. Radiographic evaluation included periapical healing, apical closure, root lengthening, and dentinal wall thickening. There was no apical closure, root lengthening, and dentinal wall thickening in Group 1 as compared to other groups which was kept as a control group.
Youssef et al., 2022 (21)	International Endodontic Journal	RCT	10	2	BC, PRF	Clinical Outcomes: There was a significant difference between the tooth sensibility readings at baseline, 6-month and 12-month follow-up time points. Radiological findings: There was a significant increase in periradicular healing in both groups at 6 and 12 months, compared to that at baseline, with no significant difference between the studied groups after 12 months
Hongbing et al., 2018 (27)	BMC oral health	Retrospective controlled cohort study	10	2	BC, PRF	PRF achieved comparable outcomes to BC in terms of Clinical outcomes: Clinical sign and symptom resolution. Radiological findings: Periapical lesion healing and root maturation in RET
Nagaveni et al., 2020 (29)	International Journal of paediatric dentistry	Case Report	2 teeth	2	BC, PRF	Clinical outcome: Both teeth showed a negative response to percussion and palpation tests but a positive response to cold and electric pulp tests. Radiological findings the tooth treated with PRF exhibited comparatively faster root lengthening, complete closure of the root apex, more thickening of the root dentinal walls, and narrowing of root canal space compared to conventionally revascularized tooth.
Uppala et al., 2020 (16)	European Journal of Molecular & Clinical Medicine	RCT	24 teeth	3	BC, PRF, Collagen	Clinical outcomes: Clinical findings revealed varied healing outcomes in different age groups, with PRF, blood clot, and collagen treatments. Root lengthening outcomes varied too. Apical closure showed different distributions. Radiological findings: no significant differences were found among groups in healing, root lengthening, apical closure, and dentinal wall thickening, highlighting comparable outcomes.
Yang et al., 2023 (28)	Australian Endodontic Journal	Retrospective study	121 teeth	2	CGF, BC	Clinical outcomes: In a study of 121 teeth from 107 children, 53 were treated with CGF and 68 with BC. Overall success rate was 91.74% over 23.15 months. Success rates were similar between CGF (86.79%) and BC (95.59%) groups, but teeth with developmental anomalies had higher success (98.39%) than those with trauma (84.31%). Radiological findings: Both CGF and BC groups showed many teeth with a score 2, indicating improved root characteristics. Scores did not differ significantly between the CGF and BC groups.
Ramachandran et al., 2021 (22)	Journal of Conservative Dentistry	Preliminary study	40	2	PRP, BC	Clinical outcomes: None of the groups tested positive for vitality Radiological findings: PRP showed increased RRA, although it was statistically non-significant

PP: Platelet pellet, BC: Blood Clot, PRF: Plasma rich fibrin, PRP: platelet-rich plasma, RRA: Radiographic root area, RCA: radiographic canal area, CBCT: cone beam computed tomography, LPRF: Leukocyte- and Platelet-rich fibrin, REP: Regenerative endodontic procedure, PBH: Periapical bone healing, PR: Periapical radiograph, PLGA: Poly(lactic-co-glycolic acid), MTA: Mineral trioxide aggregate, RET: Regenerative endodontic therapy, CGF: Concentrated growth factor.

**Table 3:** GRADE.

Certainty assessment							No. of patients		Effect		Certainty	Importance
No. of studies	Study design	Risk of Bias	Inconsistency	Indirectness	Imprecision	Other considerations	success score among control & experimental groups	placebo	Relative (95% CI)	Absolute (95% CI)		
Success Score (BC versus PRP)												
5	randomized trials	serious^a^	not serious	not serious	not serious	none	70/77 (90.0%)	72/83 (86.7%)	RR 1.01 (0.95 to 1.08)	9 more per 1,000 (from 43 fewer to 69 more)	ѳѳѳο Moderate	CRITICAL
Complete Apical Closure (Bc versus PRP)												
3	randomized trials	serious^b^	not serious	not serious	not serious	Strong Association	32/43 (74.4%)	30/46 (65.2%)	RR 1.01 (0.78 to 1.70)	98 more per 1,000 (from 143 fewer to 457 more)	ѳѳѳѳHigh	CRITICAL
Complete Apical Closure (BC versus PRF)												
4	randomized trials	not serious	not serious	not serious	not serious	Strong Association	50/60 (83.3%)	43/56 (76.8%)	RR 1.10 (0.95 to 1.27)	77 more per 1,000 (from 38 fewer to 207 more)	ѳѳѳѳ High	CRITICAL
Positive Vitality (BC versus PRF)												
3	randomized trials	not serious	not serious	not serious	not serious	Strong Association	40/39 (102.6%)	27/35 (77.1%)	RR 0.44 (0.02 to 11.62)	432 fewer per 1,000 (from 756 fewer to 1,000 more)	ѳѳѳѳHigh	CRITICAL
Positive Vitality (BC v/s PRP)												
5	randomized trials	serious^c^	serious^d^	not serious	not serious	Strong Association	46/77 (59.7%)	46/83 (55.4%)	RR 1.09 (0.69 to 1.72)	50 more per 1,000 (from 172 fewer to 399 more)	ѳѳѳο Moderate	CRITICAL
Healing Response (BC v/s PRF)												
2	randomized trials	not serious	serious^e^	not serious	serious^f^	Strong association	14/19 (73.7%)	11/19 (57.9%)	RR 1.46 (0.38 to 5.59)	266 more per 1,000 (from 359 fewer to 1,000 more)	ѳѳѳο Moderate	CRITICAL

CI: confidence interval; RR: risk ratio Explanation a. since one of the studies is having a high risk of bias. b. since one among three studies has a high risk of bias. c. since one of the studies is having a high risk of bias among 5 studies . d. since the I2 value is 73%. e. the value of I square is 76%. f. Since, wide confidence interval.

## Data Availability

The datasets used and/or analyzed during the current study are available from the corresponding author.
